# Prevalence of Low Muscle Mass in the Computed Tomography at the Third Lumbar Vertebra Level Depends on Chosen Cut-Off in 200 Hospitalised Patients—A Prospective Observational Trial

**DOI:** 10.3390/nu14163446

**Published:** 2022-08-22

**Authors:** Arabella Fischer, Noemi Kiss, Valerie-Anna Rudas, Kristina Nieding, Cecilia Veraar, Isabel Timmermann, Konstantin Liebau, Maximilian Pesta, Timo Siebenrock, Martin Anwar, Ricarda Hahn, Anatol Hertwig, Jonas Brugger, Helmut Ringl, Dietmar Tamandl, Michael Hiesmayr

**Affiliations:** 1 Department of Anaesthesia, Intensive Care Medicine and Pain Medicine, Division of Cardiothoracic and Vascular Anaesthesia and Intensive Care Medicine, Medical University of Vienna, 1090 Vienna, Austria; 2 Department of Health Economics, Center for Public Health, Medical University of Vienna, 1090 Vienna, Austria; 3 Center for Medical Statistics, Informatics and Intelligent Systems, Medical University of Vienna, 1090 Vienna, Austria; 4 Department of Biomedical Imaging and Image-Guided Therapy, Medical University of Vienna, 1090 Vienna, Austria

**Keywords:** low muscle mass, low skeletal muscle area, sarcopenia, computed tomography, body composition

## Abstract

Measuring skeletal muscle area (SMA) at the third lumbar vertebra level (L3) using computed tomography (CT) is increasingly popular for diagnosing low muscle mass. The aim was to describe the effect of the CT L3 cut-off choice on the prevalence of low muscle mass in medical and surgical patients. Two hundred inpatients, who underwent an abdominal CT scan for any reason, were included. Skeletal muscle area (SMA) was measured according to Hounsfield units on a single CT scan at the L3 level. First, we calculated sex-specific cut-offs, adjusted for height or BMI and set at mean or mean-2 SD in our population. Second, we applied published cut-offs, which differed in statistical calculation and adjustment for body stature and age. Statistical calculation of the cut-off led to a prevalence of approximately 50 vs. 1% when cut-offs were set at mean vs. mean-2 SD in our population. Prevalence varied between 5 and 86% when published cut-offs were applied (*p* < 0.001). The adjustment of the cut-off for the same body stature variable led to similar prevalence distribution patterns across age and BMI classes. The cut-off choice highly influenced prevalence of low muscle mass and prevalence distribution across age and BMI classes.

## 1. Introduction

In the last decade, a boom in computed tomography (CT) studies that analyse muscle mass could be observed. Typically, total skeletal muscle area (SMA) at the third lumbar vertebra level (L3) is measured on the CT scan as it highly correlates with whole-body muscle mass [1,2]. Low muscle mass on the CT at L3 was associated with higher mortality, longer hospital length of stay or higher infection rates in patients with cancer [3], cardiovascular [4] or gastrointestinal [5] disease, critical illness [6] and before or after surgery [7]. However, the cut-off for low muscle mass on the CT at L3 has not been consistently defined. 

First, statistical calculation of the cut-offs was different: cut-offs were calculated either as mean-2 SD [8], median [9], a percentile [10,11,12] or as a predictor for a cut-off of another imaging method (dual-energy X-ray absorptiometry (DXA) [13]), as a predictor for wound complication [14], likelihood of tumour resection [15], mortality [3] or length of stay [16]. Second, adjustment of the cut-offs was different: sometimes, SMA was non-adjusted and expressed in cm^2^ [6,8]. Most often, SMA was divided by squared height and expressed in cm^2^/m^2^ [13,17]. Other times, SMA was divided by BMI and expressed in cm^2^/(kg/m^2^) [9,18]. Cut-offs were given for each sex, sometimes even for different age and BMI groups [10,16]. Cut-off values given for different age groups decreased with higher age [10,16]. When choosing two CT scans of our study population, SMA and SMA/BMI were higher in the younger than older patient. Yet, SMA/height^2^ was higher in the older than younger patient. Similarly, a DXA study showed that the prevalence of low appendicular skeletal muscle mass (ASM) varied between 5% and 75% depending on the adjustment of ASM by either height^2^, weight or BMI [19].

For statistical calculation of the cut-off, the European Working Group on Sarcopenia (EWGSOP) recommended taking the mean-2 SD of healthy young adults [20]. Meanwhile, the International Working Group on Sarcopenia (IWGS) recommended taking the 20th percentile of healthy young adults [21]. For adjustment of the cut-off, the Foundation for the National Institutes of Health (FNIH) Sarcopenia project recommended adjusting DXA appendicular lean mass (ALM) by BMI [22,23], because it was most associated with weakness [23]. The International Working Group on Sarcopenia (IWGS) and the Asian Working Group for Sarcopenia (AWGS) recommended adjusting DXA ALM by squared height [21,24]. It therefore remains unclear which definition of which guideline should be followed.

The study’s aim was to determine the effect of the statistical calculation and the adjustment of the cut-off on the prevalence of low muscle mass in a mixed hospitalised population including medical and surgical patients. We used two types of cut-offs for the CT L3 SMA. First, we calculated new sex-specific cut-offs, which were set at the mean or mean-2 SD of our patient population and either non-adjusted or adjusted for height^2^ or BMI. Second, we applied previously published cut-offs, which highly differed in terms of statistical calculation, adjustment for body stature and subgroups (sex, BMI and age).

## 2. Materials and Methods

### 2.1. Study Design and Population

This prevalence analysis was part of the USVALID prospective observational trial (clinicaltrials.gov identifier: NCT03160222), which was performed at the Medical University of Vienna from 2017 to 2019 [25]. Adult surgical or medical inpatients, who underwent an abdominal CT scan for any clinical reason, were included (Table 1). Ethics approval was obtained from the Ethics Committee of the Medical University of Vienna. The study was conducted in accordance with the Declaration of Helsinki.

### 2.2. Computed Tomography Selection Criteria

We analysed a single CT contrast-enhanced scan at the level of the third lumbar vertebra L3 where both transverse processes were visible. Measurement was carried out semiautomatically according to Hounsfield unit values between −29 and 150 HU. Further details about the computed tomography selection criteria were previously published [25].

### 2.3. Selection of Cut-Offs for Low Muscle Mass

First, we adjusted SMA for different body stature variables (SMA, SMA/height^2^, SMA/BMI). For all adjusted SMA variables, we set sex-specific cut-offs at the mean and mean-2 SD of our study population (Table 2). Second, we selected 9 previously published cut-offs for low muscle mass, which we applied in our study population of 200 patients. The published cut-offs were different in several aspects (Table 3): they were either non-adjusted in cm^2^ [8,10] or adjusted for squared height (cm^2^/m^2^) [3,8,10,13,16,17] or BMI (cm^2^/kg/m^2^) [9]. The cut-offs were defined for subgroups of sex [3,8,9,10,13,16,17], age [16] and/or BMI [10]. They were statistically calculated as mean-2 SD [8], median [9], 5th percentile [10] or predicted low muscle mass of another reference method (DXA) [13], mortality [3,17] or hospital length of stay [16]. They were defined in patients with respiratory or gastrointestinal cancer [3,13,16,17], healthy subjects undergoing routine CT health examinations [9] or healthy kidney donor candidates [8,10]. Ethnicity was only reported in 3 studies and was Caucasian [10,13] or Asian [9]. Prevalence of low muscle mass in the selected published studies ranged from 5 to 53% (Table 3).

### 2.4. Statistical Analysis

Variables were expressed as mean ± SD or median (IQR), as appropriate. Differences in continuous variables between sexes were described with independent t-tests. SMA values were correlated to age, sex, height and BMI by calculating the coefficient of determination R^2^. Relative and absolute prevalence of low muscle mass was assessed in our study population according to the cut-offs, set at the mean or mean-2 SD in our study population and to the published cut-offs. Differences in prevalence number were calculated with one-sample chi-square tests in each sex. The prevalence distribution was described across age and BMI classes. Bar plots illustrate prevalence data. A two-sided significance level of 0.05 was applied for all tests. Analysis and graphs were carried out in R version 3.6.1.

## 3. Results

### 3.1. Description of Study Population and CT Scans

Two hundred patients were included in the USVALID study. The CONSORT flow diagram was previously published [25]. Baseline characteristics are shown in Table 1. Forty four percent of the patients had a malignant tumour (Table 1). SMA, SMA/height^2^ and SMA/BMI were higher in men than in women (*p* < 0.001) (Table 2). All SMA variables were normally distributed in our study population. CT L3 SMA positively correlated with height and BMI with an R^2^ of 0.39 and 0.15, respectively (*p* < 0.001). CT L3 SMA decreased with age only in women (R^2^ = 0.12, *p* = 0.001) but not in men (R^2^ = 0.00, *p* = 0.68). BMI (R^2^ = 0.04, *p* = 0.02) significantly increased with age in men. Height (R^2^ = 0.07, *p* = 0.02) significantly decreased with age in women. 

### 3.2. Diagnosis of Low Muscle Mass in Two Selected Patients

The two selected CT scans depict a 51-year-old, 160 cm short and a 31-year-old, 197 cm tall male patient (Table 4). The older, shorter patient had an above-average BMI of 36.3 compared to 21.9 kg/m^2^ in the younger, taller patient. The older patient had a larger CT area of 940 than 592 cm^2^ in the younger patient. The older and younger both had an above-average SMA of 151 and 163 cm^2^, respectively. However, the younger patient, because he was very tall, had a below-average SMA/height^2^ of 42.0 compared to 58.8 cm^2^/m^2^ in the older patient. On the other hand, the older patient had a below-average value of SMA/BMI compared to the younger patient (Table 4A). According to nine previously published cut-offs, two cut-offs diagnosed low muscle mass in the older patient and five in the younger patient (Table 4B).

### 3.3. Statistical Calculation of the Cut-Off Influenced Prevalence Number

When the cut-offs for SMA, SMA/height^2^ and SMA/BMI were set at the mean values of our study population, prevalence of low muscle mass ranged from 50–55% in men and 50–54% in women. This was because all SMA variables were normally distributed in both sexes of our study population. Therefore, the mean was close to the median, explaining a prevalence of around 50%. When our cut-offs were set at mean-2 SD, prevalence ranged from 0–2% in both sexes.

**Figure 1:**
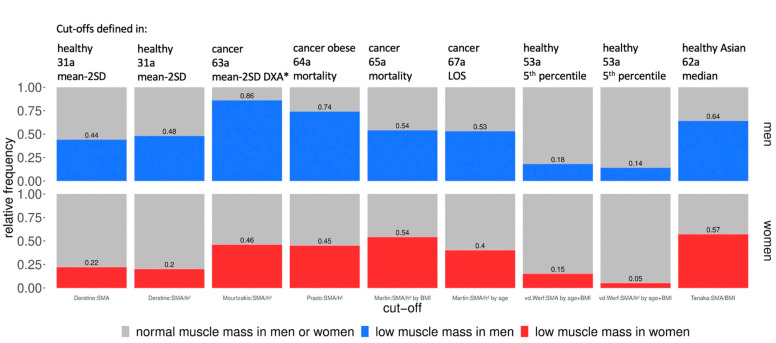
Relative prevalence of low muscle mass in our study population (n = 200) according to previously published cut-offs. * of a healthy young (29a) population; SMA: skeletal muscle area; h^2^: height^2^.

Prevalence according to the published cut-offs was highly variable from 14–86% in men (*p* < 0.001) and 5–57% in women (*p* < 0.001) (Figure 1). Prevalence was higher in men than in women (Figure 1). When the published cut-offs defined as mean-2 SD of healthy young subjects were used, prevalence in our study population was 44–48% in men and 20–22% in women. When published cut-off values of an old population were established in relation to the DXA reference values at mean-2 SD of a healthy young population, the prevalence in our study population was 86% in men and 46% in women. When published cut-offs, specifically for cancer patients and defined in relation to mortality or length of stay were used, the prevalence in our study population was 53–74% in men and 40–54% in women. When published cut-offs defined as fifth percentile in healthy subjects were used, prevalence in our study population was 14–18% in men and 5–15% in women. Finally, when published cut-offs, defined as the median of a healthy Asian population were used, the prevalence in our study population was 64% in men and 57% in women (Figure 1).

### 3.4. Adjustment of the Cut-Off-Influenced Prevalence Distribution Pattern across Age Classes

**Figure 2:**
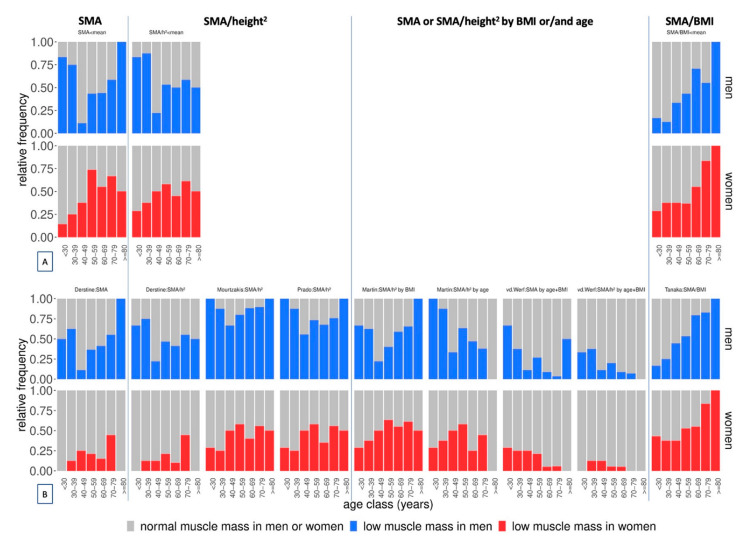
Relative prevalence of low muscle mass in our study population (n = 200) across age classes according to (**A**) cut-offs set at the mean of our study population or to (**B**) previously published cut-offs. SMA: skeletal muscle area; h^2^: height^2^.

When non-adjusted SMA or SMA/height^2^ cut-offs were used, prevalence in men was u-shaped across age classes (Figure 2). When SMA/height^2^ cut-offs were additionally defined by BMI, the u-shaped pattern remained (Figure 2). However, when SMA or SMA/height^2^ cut-offs were also defined by age, prevalence decreased with age. When SMA/BMI cut-offs were used, an increasing pattern was seen across age classes in both sexes (Figure 2).

### 3.5. Adjustment of the Cut-Off-Influenced Prevalence Distribution Pattern across BMI Classes

**Figure 3:**
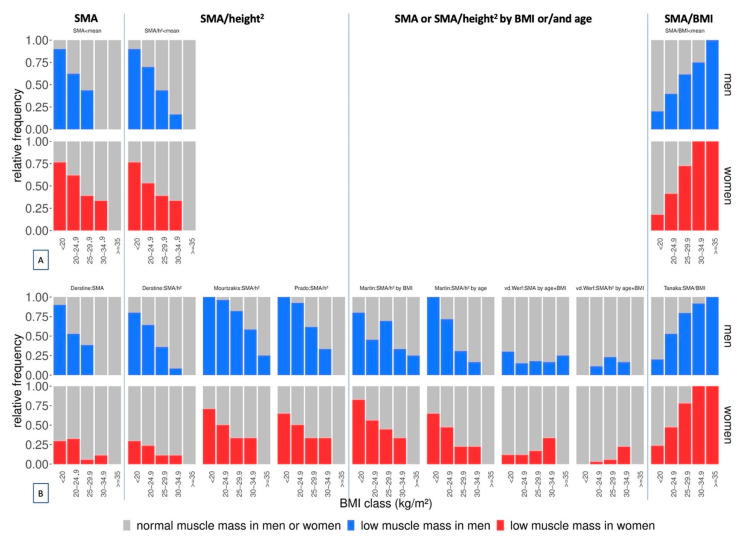
Relative prevalence of low muscle mass in our study population (n = 200) across BMI classes according to (**A**) cut-offs set at the mean of our study population or to (**B**) previously published cut-offs. SMA: skeletal muscle area; h^2^: height^2^.

When non-adjusted SMA or SMA/height^2^ cut-offs were used, prevalence decreased with higher BMI in both sexes (Figure 3). When SMA/height^2^ cut-offs were also defined by BMI or age, the decreasing pattern remained (Figure 3). However, when SMA/BMI cut-offs were used, prevalence increased with higher BMI (Figure 3).

The distribution of absolute prevalence is shown in the Supplementary Information (Figures S1 and S2). Individual CT values and diagnosis of low muscle mass in patients below 30 and above 80 years are shown in the Supplementary Information (Table S1).

## 4. Discussion

The cut-off choice had a tremendous impact on the prevalence of low muscle mass in the CT at the L3 level. Statistical calculation of the cut-off led to a prevalence of approximately 50% vs. 1% when cut-offs were set at mean vs. mean-2 SD in our study population. When previously published cut-offs were applied, prevalence varied between 5% and 86%. The adjustment of the cut-off for the same body stature variable led to similar prevalence distribution patterns across age and BMI classes.

### 4.1. Prevalence of Low Muscle Mass in Men vs. Women

All published cut-offs showed a higher prevalence of low muscle mass in men compared to women in our study population but not always in the original, published populations (Table 3). The proportion of females with low muscle mass in our study population (41%) was similar to those in the previously published studies (39–59%) (Table 3). The question arises whether men are actually at higher risk for low muscle mass than women or if this is related to the calculation of the published cut-offs. Interestingly, Janssen showed that the age-associated decrease in whole-body MRI muscle mass from 45 years onwards was steeper in men than in women [29]. Thus, it may be possible that men are at higher risk for developing low muscle mass.

### 4.2. Statistical Calculation of the Cut-Off-Influenced Prevalence Number

When applying our own cut-offs, their statistical calculation at mean and mean-2 SD obviously explained the different prevalence of 50% and 1% in our normally distributed population. When applying published cut-offs, their statistical calculation also explained the prevalence number in our population. When age and BMI were accounted for, published cut-offs defined at the fifth percentile of healthy subjects led to the lowest prevalence numbers between 5 and 18% in our hospitalised population. When age and BMI were not accounted for, published cut-offs at mean-2 SD of a young and healthy population led to a higher prevalence number in our middle-aged, hospitalised population. Applying cut-offs calculated in a muscular, young, healthy group in an older, hospitalised population must obviously lead to a higher prevalence when age is not accounted for. When applying cut-offs defined in relation to mortality or length of stay in published cancer populations, prevalence was between 40% and 74% in our mixed population including 44% of cancer patients. When applying cut-offs defined as median of an Asian, healthy population, prevalence was higher than 50% in our Caucasian population. 

### 4.3. Adjustment of the Cut-Off-Influenced Prevalence Distribution Pattern across Age Classes

When non-adjusted SMA or SMA/height^2^ cut-offs were used, prevalence was u-shaped in men across age classes. Looking at the individual SMA or SMA/height^2^ values of the very young and very old patients (“the borders of the u”) helps to understand the u-shaped pattern: the individual SMA or SMA/height^2^ values themselves are not adjusted for the overall low BMI in the young and the older age in the old (Supplementary Information, Table S1). This may lead to a higher prevalence observed in the low and high extremes of age when non-adjusted SMA or SMA/height^2^ cut-offs were used. This justifies the importance of accounting for BMI and age when diagnosing low muscle mass. The u-shaped pattern across age classes remained when the SMA/height^2^ cut-off was defined by BMI. An increasing pattern was observed when SMA/BMI cut-offs were used. This may justify the need to account not only for BMI but also for age. When the SMA/height^2^ cut-off was defined by age, a decreasing pattern was seen across age classes. The adjustment for age led to lower cut-offs in higher age classes (Table 3). It makes sense to compare the muscle mass of an 80-year-old man to men of a comparable age and not to young 20-year-olds. Otherwise, all 80-year-olds would be diagnosed with low muscle mass, which actually occurred according to nearly all non-age-specific cut-offs in men. The non-age-specific cut-offs actually followed the recommendations of both the EWGSOP and IWGS to compare muscle mass to a reference value of the young and healthy population [20,21]. We think that a discussion of whether similar muscle mass can be assumed in younger and older patients is warranted.

### 4.4. Adjustment of the Cut-Off-Influenced Prevalence Distribution Pattern across BMI Classes

When non-adjusted SMA or SMA/height^2^ cut-offs were used, prevalence decreased with higher BMI in both sexes. This is because SMA increases with higher BMI. The higher the BMI value, the higher the SMA was. This result questions the notion of sarcopenic obesity. Heavier people may simply need more muscle mass to carry themselves around. Obese people may actually have a lower risk for low muscle mass. When SMA/BMI cut-offs were used, prevalence increased with higher BMI. This is because the double visualisation of BMI (adjusting SMA for BMI and looking graphically at distribution across BMI classes) may be misleading.

### 4.5. Prevalence Numbers of Low Muscle Mass in the Literature

Prevalence of low muscle mass on the CT at L3 in two recent publications was from 36–50% in patients with cancer [30] and 27–45% in patients with cirrhosis [31]. A systematic review in cancer patients concluded that prevalence of low muscle mass was irrespective of the cut-off used [30]. However, they included only studies with cut-offs that adjusted for height^2^ [30]. Moreover, 55% of the included studies used either the CT cut-offs from Prado, 2008, or Martin, 2013, both of which were derived from the same initial population cohort (Table 3) [3,17].

To the best of our knowledge, we are the first showing that the CT cut-off choice has a tremendous impact on the prevalence of low muscle mass: varying between 5% and 86% depending on the statistical definition and the adjustment of the cut-off. Only Kim already described a similar variation in prevalence of low ASM measured by DXA between 5% and 75% depending on the adjustment of the cut-off for height^2^, weight or BMI [19].

### 4.6. Limitations and Strengths

One may criticise that we applied published cut-offs issued from various study populations with different ages, BMIs and ethnicities (Table 3). We carried that out on purpose to reflect many previous publications, which often reapplied cut-offs initially defined in a completely different study population. Prado’s highly cited and reapplied cut-off was initially defined in a probably mostly Caucasian respiratory and gastrointestinal cancer population with a mean age of 64 years and a mean BMI of 34 kg/m^2^ [17] but reapplied for diagnosis of low muscle mass on the CT at L3 in many other study populations including patients with pancreatic [32,33] or breast cancer [34], medical patients with cirrhosis [5] and vascular surgery patients [35] with a mean age varying between 48 and 66 years [5,32,33,34,35], with a mean BMI varying between 22 and 29 kg/m^2^ [5,32,33,34,35] and with Caucasian [32], Asian [33] or multiple ethnicities [5,35]. In our current publication, we did not aim for completeness but for high diversity of all cut-off adjustments for CT L3 SMA. We are currently working on a systematic review and meta-analysis to give an overview of over 100 published CT L3 SMAs (PROSPERO 2020, CRD42020206919) [36]. 

## 5. Conclusions

In this prospective study, we have shown for the first time that prevalence of low SMA in the CT at L3 tremendously depended on the applied cut-off. The statistical calculation and the adjustment of the cut-off for body stature variables, for subgroups of sex, age or BMI significantly influenced the prevalence number of low muscle mass and prevalence distribution pattern across age and BMI classes. Both age and BMI are important factors to account for when diagnosing low muscle mass.

## Figures and Tables

**Table 1:** Baseline characteristics of the study population (n = 200).

Characteristic	All (n = 200)	Male (n = 118)	Female (n = 82)
Age (years)	61.3 (51.0–70.1) (19–86)	63.6 (51.4–71.3) (19–86)	58.9 (45.8–68.8) (21–85)
Weight (kg)	73.9 ± 16.0 (41–118)	79.1 ± 14.0 (47–110)	66.3 ± 15.8 (41–118)
Height (cm)	172.0 ± 9.4 (148–197)	177.1 ± 7.3 (160–197)	164.6 ± 6.8 (148–183)
BMI (kg/m^2^)	24.9 ± 4.8 (16.2–42.0)	25.2 ± 4.4 (16.5–38.3)	24.5 ± 5.4 (16.2–42.0)
Functional comorbidity index (FCI) (points) [26]	2 (1–3) (0–10)	2 (1–3) (0–7)	2 (1–4) (0–10)
Kidney injury	21 (10.5)	14 (11.9)	7 (8.5)
Current presence of malignant tumour	88 (44)	48 (40.7)	40 (48.8)
Surgical wards	135 (67.5)	77 (65)	58 (70.7)
General surgery	71 (35.5)	44 (37.3)	27 (32.9)
Urology	35 (17.5)	23 (19.5)	12 (14.6)
Gynaecology	13 (6.5)	-	13 (15.9)
Cardiac surgery	8 (4.0)	4 (3.4)	4 (4.9)
Vascular surgery	5 (2.5)	4 (3.4)	1 (1.2)
Orthopaedic surgery	2 (1.0)	1 (0.8)	1 (1.2)
Thoracic surgery	1 (0.5)	1 (0.8)	0 (0)
Medical wards	65 (32.5)	41 (34.7)	24 (29.3)
Gastroenterology	41 (20.5)	27 (22.9)	14 (17.1)
Oncology	11 (5.5)	4 (3.4)	7 (8.5)
Nephrology	6 (3.0)	4 (3.4)	2 (2.4)
Cardiology	5 (2.5)	4 (3.4)	1 (1.2)
Haematology	2 (1.0)	2 (1.7)	0 (0)
Time between CT and ultrasound, hours	22 (5–28) (1–48)	21 (5–27) (1–48)	22 (6–29) (1–48)
Clinical presence of peripheral oedema	41 (20.5)	24 (20.3)	17 (20.7)
Patients with surgery prior to ultrasound examination	73 (36.5)	43 (36.4)	30 (36.6)
Time between prior surgery and ultrasound, days	5 (2–10) (0–59)	5 (2–11) (0–40)	4 (2–9) (0–59)
Hospital length of stay, days	13 (6–23) (1–174)	15 (6–26) (1–174)	12 (6–23) (1–96)
Hospital mortality	5 (2.5)	3 (2.5)	2 (2.4)
PANDORA score (points) [27]	26.5 (19–34)(2–56)	26 (20–33.8) (6–54)	27.5 (19–35) (2–56)

Data are indicated as n (%), median (IQR) (range) or mean ± SD (range), as appropriate.

**Table 2:** CT measurements (n = 200).

	All (n = 200)		Male (n = 118)		Female (n = 82)		
CT measurements	mean	SD	mean	SD	mean	SD	P
SMA (cm^2^)	131.9	29.5	148.3	23.7	108.3	19.4	<0.001
SMA/height^2^ (cm^2^/m^2^)	44.3	8.0	47.3	7.6	40.0	6.3	<0.001
SMA/BMI (cm^2^/(kg/m^2^))	5.4	1.2	6.0	1.0	4.6	1.0	<0.001

SMA: skeletal muscle area (cm^2^); *p* values are presented for differences between men and women (independent *t*-test).

**Table 3:** Selected published cut-offs.

Publication	Cut-Off Adjustment	Cut-Off Values Defined for Subgroups									Cut-Off Calculation	Study Population	Mean Age	Prevalence of Low Muscle Mass
													Mean BMI	
													Ethnicity	
Derstine, 2018 [8]	SMA	Male: <144.3 cm^2^ Female: <92.2 cm^2^									Mean-2 SD of a healthy, young population	n = 727 (410 female) healthy kidney donor candidates for CT at L3 level	31 ± 6 years BMI: ~27 ± 16 NR (study conducted in the USA)	Male: NR Female: NR
Derstine, 2018 [8]	SMA/height^2^	Male: <45.4 cm^2^/m^2^ Female: <34.4 cm^2^/m^2^									Mean-2 SD of a healthy, young population	n = 727 (410 female) healthy kidney donor candidates for CT at L3 level	31 ± 6 years BMI: ~27 ± 16 NR (study conducted in the USA)	Male: NR Female: NR
Mourtzakis, 2008 [13]	SMA/height^2^	Male: < 55.4 cm^2^/m^2^ Female: < 38.9 cm^2^/m^2^									Equation to predict DXA cut-offs [28] for low muscle mass	n = 31 (12 female) non-small cell lung or colorectal cancer patients	63 ± 10 years BMI: 26.9 ± 6.2 96% Caucasian	Male: NR Female: NR
Prado, 2008 [17]	SMA/height^2^	Male: <52.4 cm^2^/m^2^ Female: <38.5 cm^2^/m^2^									Optimal stratification related to mortality	n = 250 (114 female) respiratory or gastrointestinal cancer patients with BMI ≥ 30	64 ± 10 years BMI: 34.4 ± 4.4 NR (study conducted in Canada)	Male: 21% Female: 9%
Martin, 2013 [3]	SMA/height^2^	Male with BMI < 25: 43 cm^2^/m^2^ Male with BMI ≥ 25: 53 cm^2^/m^2^ Female (all BMI): <41 cm^2^/m^2^									Optimal stratification related to mortality	n = 1473 (645 female) respiratory or gastrointestinal cancer patients (same initial patient cohort as Prado’s study [17])	65 ± 11 years BMI: ~25.5 NR (study conducted in Canada)	Male: 31% Female: 53%
Martin, 2018 [16]	SMA/height^2^	Age (years)	Male (cm^2^/m^2^)	Female (cm^2^/m^2^)							Generalized linear model with a negative binomial distribution related to hospital length of stay	n = 2100 (830 female) Colorectal cancer patients	67 ± 12 yearsBMI: 27.7 ± 5.6NR (study conducted in Canada and UK)	Male: NRFemale: NR
		<50	<50.6	<39.6										
		50–59	<49.3	<37.6										
		60–69	<46.8	<37.1										
		70–79	<43.4	<35.2										
		≥80	<38.7	<33.5										
van der Werf, 2018 [10]	SMA		Male				Female				Predicted 5th percentile of SMA from BMI and age in a regression equation	n = 420 (246 female) healthy kidney donors	53 ± 12 yearsBMI: 25.7 ± 3.5Caucasian	Male: 5%Female: 5%
			BMI: 17–20	BMI: 20–25	BMI: 25–30	BMI: 30–35	BMI: 17–20	BMI: 20–25	BMI: 25–30	BMI: 20–35				
		20–29 years	131.4	145.4	162.6	179.3	88.2	102.7	119.4	134.7				
		30–39 years	124.3	138.3	155.5	172.2	86.8	97.9	111.2	123.7				
		40–49 years	117.1	131.2	148.3	165.0	85.1	93.1	102.9	112.3				
		50–59 years	109.8	123.8	141.0	157.7	83.0	88.2	94.4	100.6				
		60–69 years	102.3	116.4	133.6	150.3	80.7	83.1	85.9	88.4				
		70–79 years	94.8	108.8	126.0	142.7	78.0	78.0	77.3	75.9				
van der Werf, 2018 [10]	SMA/height^2^		Male				Female				Predicted 5th percentile of SMA/height2 from BMI and age in a regression equation	n = 420 (246 female) healthy kidney donors	53 ± 12 yearsBMI: 25.7 ± 3.5Caucasian	Male: 5%Female: 5%
			BMI: 17–20	BMI: 20–25	BMI: 25–30	BMI: 30–35	BMI: 17–20	BMI: 20–25	BMI: 25–30	BMI: 20–35				
		20–29 years	37.4	42.5	48.7	54.8	28.5	33.7	39.6	45.1				
		30–39 years	35.9	41.0	47.2	53.3	28.7	32.8	37.6	42.2				
		40–49 years	34.3	39.4	45.6	51.7	28.8	31.8	35.6	39.2				
		50–59 years	32.7	37.7	43.9	50.0	28.7	30.9	33.5	36.1				
		60–69 years	31.0	36.1	42.3	48.4	28.5	29.9	31.4	32.9				
		70–79 years	29.3	34.4	40.6	46.7	28.2	28.8	29.3	29.5				
Tanaka, 2020 [9]	SMA/BMI	Male: <6.309 cm^2^/kg/m^2^ Female: <4.66 cm^2^/kg/m^2^									Median of study population	n = 632 (279 female) employees undergoing CT health examinations	~62 years BMI: ~24 Asian	Male: 50% Female: 50%

NR: not reported; BMI in kg/m^2^; DXA: dual-energy X-ray absorptiometry.

**Table 4:** Diagnosis of low muscle mass in two selected study patients according to (**A**) cut-offs set at the sex-specific mean of our study population or (**B**) previously published cut-offs.

	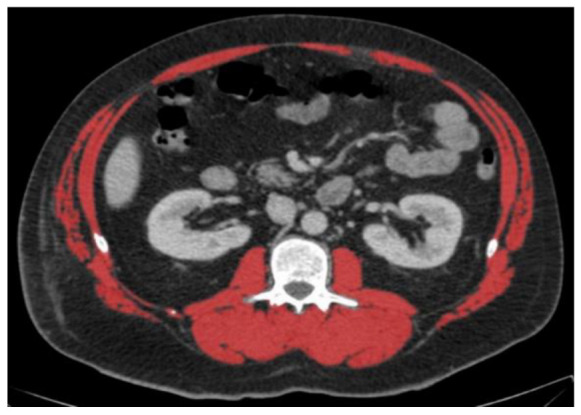	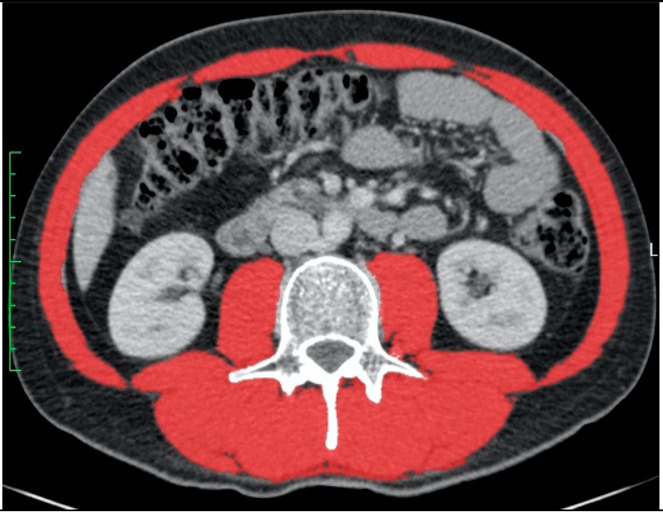	All male patients (n = 118) *
Sex	Male	Male	Male
Age (years)	51	31	63.6 (51.4–71.3)
Height (cm)	160	197	177.1 ± 7.3
Weight (kg)	93	85	79.1 ± 14.0
BMI (kg/m^2^)	36.3	21.9	25.2 ± 4.4
CT area (cm^2^)	939.8	592.4	749.8 ± 187.6
A: Diagnosis of low or normal muscle mass according to sex-specific cut-offs set at the mean of our study population			
SMA (cm^2^)	150.6 (normal)	162.9 (normal)	148.3 ± 23.7
SMA/height^2^ (cm^2^/m^2^)	58.8 (normal)	42.0 (low)	47.3 ± 7.6
SMA/BMI (cm^2^/(kg/m^2^))	4.1 (low)	7.4 (normal)	6.0 ± 1.0
B: Diagnosis of low or normal muscle mass according to published cut-offs for low muscle mass			
Derstine, 2018: SMA by sex [8]	Normal	Normal	
Derstine, 2018: SMA/height^2^ by sex [8]	Normal	Low	
Mourtzakis, 2008: SMA/height^2^ by sex [13]	Normal	Low	
Prado, 2008: SMA/height^2^ by sex [17]	Normal	Low	
Martin, 2013: SMA/height^2^ by sex and BMI [3]	Normal	Low	
Martin, 2018: SMA/height^2^ by sex and age [16]	Normal	Low	
van der Werf, 2018: SMA by sex, age and BMI [10]	Low	Normal	
van der Werf, 2018: SMA/height^2^ by sex, age and BMI [10]	Normal	Normal	
Tanaka, 2020: SMA/BMI by sex [9]	Low	Normal	

* Mean ± SD or median (IQR) are indicated as appropriate.

## Data Availability

Part of the data presented in this study is available in Table S1.

## Supplementary Materials

The following supporting information can be downloaded at: https://www.mdpi.com/article/10.3390/nu14163446/s1, Figure S1: Absolute prevalence of low muscle mass in our study population (n = 200) across age classes according to (A) cut-offs set at the mean of our study population or to (B) previously published cut-offs; Figure S2: Absolute prevalence of low muscle mass in our study population (n = 200) across BMI classes according to (A) cut-offs set at the mean of our study population or to (B) previously published cut-offs; Table S1: Individual CT values and diagnosis of normal (=0) or low (=1) muscle mass according to cut-offs set at mean of our study population or to previously published cut-offs in patients below 30 and above or equal to 80 years.
