# Supplementary material for: Prevalence of Low Muscle Mass in the Computed Tomography at the Third Lumbar Vertebra Level Depends on Chosen Cut-Off in 200 Hospitalised Patients—A Prospective Observational Trial
Source: Nutrients. 2022 Aug 22;14(16):3446. doi: 10.3390/nu14163446 (PMC9413680; doi:10.3390/nu14163446)
Supplement: Supplementary file 1 [file nutrients-14-03446-s001.zip › nutrients-1864702-Supplementary Figures.pdf]

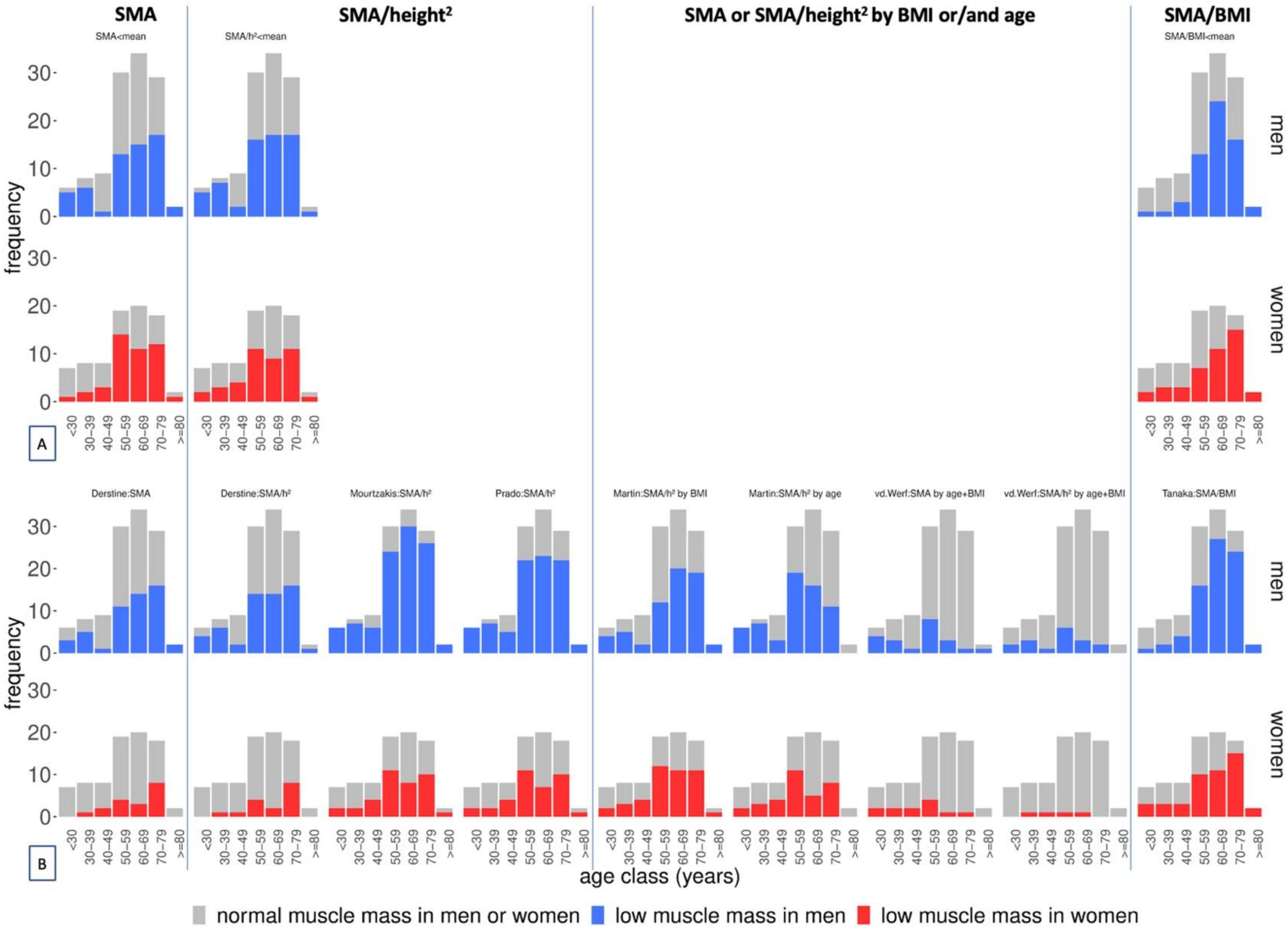

Figure S1. Absolute prevalence of low muscle mass in our study population (n=200) across age classes according to (A) cut-offs set at the mean of our study population or to (B) previously published cut-offs

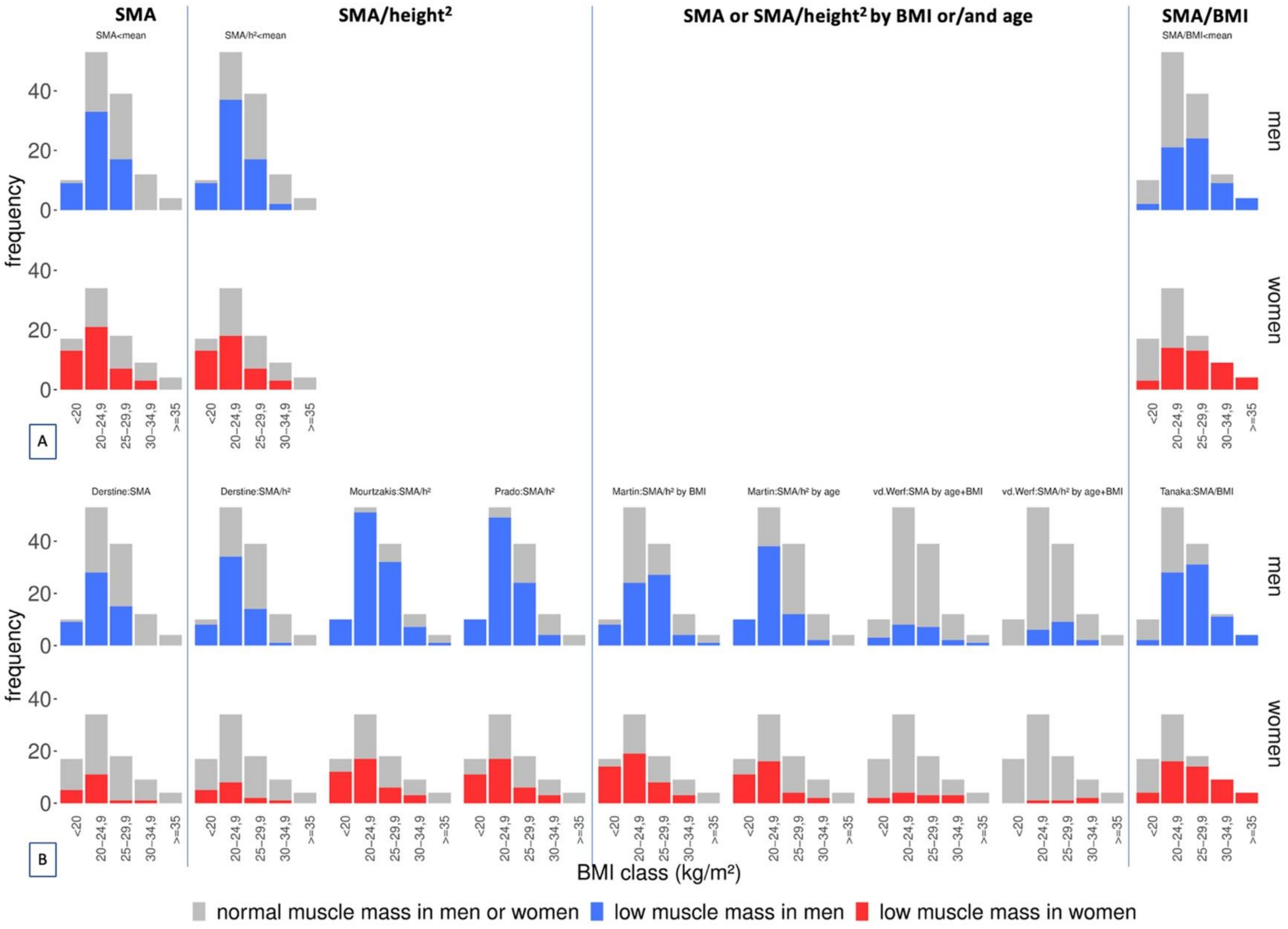

Figure S2. Absolute prevalence of low muscle mass in our study population (n=200) across BMI classes according to (A) cut-offs set at the mean of our study population or to (B) previously published cut-offs
